# Approaches for the establishment of optimized co-culture system of multiple *Trichoderma* strains for culture metabolites highly effective in cucumber growth promotion

**DOI:** 10.3389/fmicb.2022.1020077

**Published:** 2022-09-27

**Authors:** Hongyi Liu, Dazhi Hao, Yaqian Li, Xinhua Wang, Jie Chen

**Affiliations:** ^1^School of Agriculture and Biology, Shanghai Jiao Tong University, Shanghai, China; ^2^State Key Laboratory of Microbial Metabolism, Shanghai Jiao Tong University, Shanghai, China

**Keywords:** *Trichoderma*, co-culture, medium optimization, biocontrol metabolites, growth promotion metabolites, response surface methodology

## Abstract

In most cases, co-culture of *Trichoderma* and other microorganism principally takes advantage of biological control of plant diseases, which is superior to axenic culture. However, the approach to establish the optimal co-culture system of multiple *Trichoderma* strains was less studied, particularly for high production of microbial metabolites synergistically to promote plant growth and antagonistic activity against pathogens. The inoculation technique, fermentation kinetic modeling and response surface methodology were used to obtain the optimal inoculum sequence, fermentation time and co-culture nutrient formula. It was demonstrated that co-culture metabolites of *Trichoderma* strains obtained by simultaneous inoculation were more effective than those by sequence inoculation in promoting cucumber seedling growth. Furthermore, the optimal fermentation time was determined at 96–120 h by evaluating fermentation kinetic model, the activities of inhibitory potential of pathogenic *Fusarium* and cucumber seedling hypocotyl growth. Interestingly, the optimized nutrient formula was set to make co-culture metabolites of *Trichoderma* strains more effective in the plant growth promotion, which was determined through the assessment of cucumber test-tube plantlet. The components and each concentration in the optimized medium were confirmed at corn flour 16.22 g⋅L^–1^, potassium hydrogen phosphate 1.13 g⋅L^–1^, tryptophan 0.154 g⋅L^–1^, seaweed residue 30 g⋅L^–1^, ferrous sulfate heptahydrate 1 g⋅L^–1^ and ammonium sulfate 1.5 g⋅L^–1^. The hypocotyl length increased in the treatment with co-culture metabolites from the optimal medium by 2.3-fold compared with control. Thus, the results provide an optimal co-culture system of *Trichoderma* multiple strains aiming to produce high activity of metabolites in plant growth promotion.

## Introduction

*Trichoderma* is a widely distributed biocontrol fungus for plant disease, which has a broad antimicrobial spectrum and reproduces rapidly (Papavizas, 1985). It has obvious advantages in controlling soil-borne diseases in crops, such as maize, wheat, and cucumber (Papavizas, 1985; Lorito et al., 2010). The biocontrol ability of *Trichoderma* is mainly accomplished by producing antagonistic substances, spatial and nutritional competition, mycoparasitism, and inducing plant resistance (Harman, 2006; Mukherjee et al., 2013). *Trichoderma* can also promote plant growth, improve nutrient utilization efficiency, enhance plant stress resistance and bioremediate agrochemical polluted environment (Harman, 2006; Druzhinina et al., 2011). As usual, only one *Trichoderma* strain has shown a limited kind of metabolites in the axenic culture process (Keswani et al., 2013). However, in the natural farming system, a range of challenges are faced along crops growth season, particularly pathogen infection and stress environment limiting plant growth (Keswani et al., 2013). Therefore, it is significant to obtain multiple functional metabolites by the co-cultivation of *Trichoderma* consortia strains. Thus, the co-culture sourced metabolites would provide an alternative approach leading to synergistic effects of plant disease control and plant growth (Netzker et al., 2015). The recent research revealed that antagonistic activity and plant growth promotion ability of the co-culture of *Trichoderma* and other fungi and bacteria has been demonstrated to be superior compared to axenic culture if the consortia is optimized (Knowles et al., 2022). Efficient cooperation among microbes in co-culture can promote the production of various secondary metabolites, such as *Trichoderma* spp. and *Bacillus* spp. with different functions (Bertrand et al., 2014). To obtain more diversified metabolites from co-cultured *Trichoderma* strains, the effective interaction between *Trichoderma* strains in the same fermenter can be a prerequisite to positively regulate the production of known metabolites or induce the production of novel metabolites sourced from different strains. Usually, the balanced growth between strains in co-culture process depends upon many factors including the inoculation mode. Recent study reported that, in some cases, the same time inoculation of multiple strains in medium would result in interference of fast growth strain to slowly growing strains. To address the challenging problem, the sequence inoculation and optimized ratio of strains inoculation have been successfully innovated to overcome this dilemma, which could reach a balanced growth and interaction of multiple strains in the same medium aiming to produce more co-culture diverse metabolites and synergistic action of consortia (Karuppiah et al., 2019; Tang et al., 2020).

The liquid fermentation can produce substantial amino acids (alanine, aspartate, and glutamate), organic acids (glyoxylate and dicarboxylate), antagonistic chemicals (trigonelline and 5-hydroxytryptophan), plant hormones (zeatin), and other compounds (Ni et al., 2019). The component in the medium and their concentrations play an important role in the production of primary and secondary metabolites, particularly, the carbon and nitrogen components present in the medium can affect the efficiency of metabolite production (Singh et al., 2017). Traditional a-factor-a-time, Plakett–Burman design, response surface methodology has been used to optimize the composition and concentration of fermentation medium and culture conditions (Ahamed and Vermette, 2010; Sriram et al., 2011; de Rezende et al., 2020). It has been proved that *Trichoderma* can utilize a wide range of carbon and nitrogen sources, as well as inorganic salts and trace elements (Pu et al., 2013; Zhao et al., 2019).

In the present study, we combined traditional non-statistical technology and statistical technology-based experimental designs to optimize the medium for *Trichoderma* spp. co-culture substances. The one-factor-at-a-time experiments were performed to identify the medium components supposed to have influenced production. The Plakett-Burman design was conducted to screen the significant components. And central composite design, a response surface methodology, was employed to build models to evaluate the effective factors, examine their interaction and select an optimum concentration. The test tube cucumber seedling plantlet was applied to fast evaluate the quality of *Trichoderma* co-culture metabolites. Finally, we determined the optimization of co-culture medium and fermentation parameters in details for the high production of antagonism and plant growth promotion related metabolites in the co-culture process of *Trichoderma* strains.

## Materials and methods

### Fungal strains

*Trichoderma asperelloides* Z4-1 (CGMCC NO.40245), *T. harzianum* RW10569 (CGMCC NO.40246), *T. asperellum* GDFS1009 (CGMCC NO.9512), and *T. asperellum* SBW10264 (CGMCC NO.22404) was obtained from the China General Microbiological Culture Collection Center. *Fusarium oxysporum* were provided by the Laboratory of Plant Pathology, Shanghai Jiao Tong University. *Cucumis sativus* L. seeds (Shenqing NO. 1) were purchased from Shanghai Funong Seed Industry Co., Ltd.

### Inoculation technology for co-culture of *Trichoderma* spp. in the fermenter

#### Culture condition of *Trichoderma* spp.

The co-culture of *Trichoderma* composite was carried out in a 50 L fermentation tank (Guoqiang, China). The laboratory scale fermenter was equipped with a stirring speed controller (100–900 rpm), temperature controller (5–60°C), automatic pH adjustment (ammonia), and airflow (0.2 μm filter) rate adjustment were employed. Four *Trichoderma* strains were grown on potato dextrose agar (PDA) medium for 5 days at 28°C, respectively. The spores were washed with sterile water and the concentration was adjusted to 2 × 10^8^ spore/ml. A total of 0.5 mL spore suspensions of four *Trichoderma* strains were inoculated into 100 mL PD medium and incubated for 2 days at 28°C and 200 rpm as the seed culture. The four kinds of starting inoculum of *Trichoderma* strains were prepared, of which, 0.03 % (v/v) of each starting inoculum was inoculated into a 70 % fermentation medium [Corn flour 50 g/L, KH_2_PO_4_ 3.82 g/L, NaNO_3_ 1.42 g/L, (NH_4_)_2_SO_4_ 1.1 g/L, NaCl 1 g/L, MgSO_4_⋅7H_2_O 0.5 g/L, FeSO_4_⋅7H_2_O 0.0075 g/L, MnSO_4_ 0.0025 g/L, ZnSO_4_ 0.002 g/L]. The medium was kept at 28°C and pH 7, the agitation speed and airflow rate were fixed at 120 rpm and airflow 100%, respectively. During the fermentation time, the fermentation broth was analyzed by examining the culture every 24 h intervals.

#### Cucumber test tube seedling culture

Cucumber seeds with the same size and fullness were selected for surface disinfection in which seeds were soaked in 75 % alcohol for 30 s, then rinsed with sterile water three times, re-soaked in 3 % sodium hypochlorite for 8–10 min, and finally washed with sterile water for 3–4 times. The sterilized seeds were evenly placed in the Petri dish, which was coated with the sterilized double-layer filter paper that had been soaked in water. The cucumber seeds were germinated at 26°C for 3 days in the dark. The filtered *Trichoderma* co-culture broth was mixed with sterilized water in a ratio of 1:44. The germinated cucumber buds with approximate length were put into the 5 mL centrifuge tube, which was filled with 4 ml mixed solution and a rolled 3.5 mL filter paper. The radicle was pointing downward and preventing the germ from submerging in the liquid. The cucumber hypocotyl length was counted after dark culture at 26°C for 5 day.

### Selection of the optimal fermentation time for co-culture of *Trichoderma* spp.

#### Pathogen inhibition assay

*Fusarium oxysporum* was grown for 5 days at 28°C on PDA plates. Co-culture metabolites were filtered through a 0.22 μm Millex membrane (Millipore, USA) to remove living cells before use. The co-culture metabolites were mixed with PDA (50°C) at a ratio of 1:9. The plugs of mycelium were taken from the edge of the colony with a punch (diameter 0.5 cm). *F. oxysporum* plug was inoculated at the center of the PDA plate containing co-culture metabolites and cultured at 28°C for 5 days. The PDA plates without metabolites were used as control. Each treatment was replicated three times. The diameter of the colony was measured when the control plate was overgrown, and the inhibition rate was calculated as Equation (1).


(1) Inhibitionrate(%)=

$$\frac{\left(\mathrm{diameter}\,\mathrm{of}\,\mathrm{control}\,\mathrm{group}-\mathrm{diameter}\,\mathrm{of}\,\mathrm{treatment}\,\mathrm{group}\right)}{\mathrm{diameter}\,\mathrm{of}\,\mathrm{control}\,\mathrm{group}}$$

×100

#### Biomass evaluation

The biomass of the co-cultivation of four *Trichoderma* strains was evaluated by the mycelium dry weight method. The constant weight filter paper was used for filtration. After the fermentation broth was filtered, the filter paper and mycelium were dried at 85°C for 12 h, and the biomass of the mycelium were obtained by weighing the filter paper and mycelium. The spore yield of the co-cultivation of four *Trichoderma* was measured by the dilution plate coating method. The *Trichoderma* co-culture solution was diluted with sterile into a gradient of 10^–5^ to 10^–7^. A total of 0.1 ml diluent was added to the PDA plate evenly with a coating rod. The coated plates were placed on the worktable for 10 min to make the solution penetrate the medium. Then the plates were inverted and cultured at 28°C for 24 h. The *Trichoderma* colony number was counted after that. Each treatment was replicated three times.

#### Growth promotion and antimicrobial metabolites analysis

Enzyme activity was determined by the Microplate Reader (SpectraMaxi3x, Molecular Devices, USA) at room temperature. The cultures were sampled and centrifuged at 8,000 rpm and 4°C for 5 min to remove mycelia, and then the supernatant was collected, which was appropriately diluted before analyzing the activities of chitinase, β-1,3-glucanase, and acid protease. The enzyme activities were estimated using Enzyme Activity Determination Kits (Solarbio Technology, China) according to the manufacturer’s protocol. Reducing sugar was measured using Determination Kits (Leagene Biotechnology, China). Indole-3-acetic acid (IAA) and indole-3-butyric acid (IBA) was measured using ELISA Kits (Shaoxin Biotechnology, China). Results were presented as the mean of triplicate for the flask culture with standard deviations at a significance of *p* < 0.05.

#### The logistic equation for microbial growth

The Logistic equation was used to describe the biomass of the co-culture *Trichoderma* strains and it can be described as Equation (2).


(2) $$\frac{d\,X}{d\,t}=\mu_{m}\,X\,\left(1-\frac{X}{X_{m}}\right)$$

where μ_*m*_ is the maximum specific growth rate, h^–1^; *X* is the dry weight of *Trichoderma*; *X_m_*is the maximum attainable biomass concentration, g⋅L^–1^. The integrated form of Equation (2) is the following:


(3) $$X=\frac{X_{0}\,\text{exp}\,\left(\mu_{m}\,t\right)}{1-\left(X_{0}/X_{m}\right)\,\left(1-\exp\left(\mu_{m}\,t\right)\right)}$$

where *X_0_* is the initial biomass concentration (g⋅L^–1^) and t is time (h).

#### The Luedeking–Piret equation for production formation

The kinetics of product formation was based on the Luedeking–Piret’s equation (Luedeking and Piret, 2000) and it can be described as Equation (4).


(4) $$\frac{d\,P}{d\,t}=\alpha \,\frac{d\,X}{d\,t}+\beta \,X$$

X is the biomass concentration (g⋅L^–1^), α is a growth-associated constant (U⋅g^–1^) and β is a non-growth-associated constant (U⋅g^–1^⋅h^–1^). The values of α and β depend on the fermentation conditions. Substitution of Equation (2) and (3) in (4) results in the following relationship:


(5) $$\frac{d\,P}{d\,t}=\alpha \,\left[\mu_{m}\,X\,\left(1-\frac{X}{X_{m}}\right)\right]+$$

$$\beta \,\left[\frac{X_{0}\,\text{exp}\,\left(\mu_{m}\,t\right)}{1-\left(X_{0}/X_{m}\right)\,\left(1-\exp\left(\mu_{m}\,t\right)\right)}\right]$$

Equation (5) can be integrated using the initial condition *t* = 0, X = *X_0_* and P = *P_0_*, to produce the following equation:


(6) $$P-P_{0}=\alpha \,\left[\frac{X_{0}\,\exp\left(\mu_{m}\,t\right)}{1-\left(\frac{X_{0}}{X_{m}}\right)}-X_{0}\right]+$$

$$\beta \,\left[\left(\frac{X_{m}}{\mu_{m}}\right)\,\ln\left(1-\left(\frac{X_{0}}{X_{m}}\right)\,\left(1-\exp\left(\mu_{m}\,t\right)\right)\right)\right]$$

where P is the concentration of the metabolite (U⋅L^–1^), *P_0_* is the initial metabolites concentration (U⋅L^–1^) and t is time (h).

#### The Luedeking–Piret equation for glucose consumption

The classical kinetic model of was used to fit substrate uptake (Luedeking and Piret, 1959).


(7) $$-\frac{d\,S}{d\,t}=\frac{1}{Y_{G}}\,\left(\frac{d\,X}{d\,t}\right)+m_{S}\,X$$

X is the biomass concentration (g⋅L^–1^), *Y_G_* is the model predicted biomass yield coefficient on glucose (mg⋅g^–1^) and *m_S_* is the cell maintenance coefficient on glucose (mg⋅g^–1^⋅h^–1^). Substitution of Equation (2) and (3) in (7) produced the following equation:


(8) $$-\frac{d\,y}{d\,x}=\frac{1}{Y_{G}}\,\left[\mu_{m}\,X\,\left(1-\frac{X}{X_{m}}\right)\right]+m_{S}$$

$$\left[\frac{X_{0}\,\text{exp}\,\left(\mu_{m}\,t\right)}{1-\left(X_{0}/X_{m}\right)\,\left(1-\exp\left(\mu_{m}\,t\right)\right)}\right]$$

Integration of Equation (7) for the initial condition, *t* = 0, X = *X_0_* and *S* = *S_0_*, led to the following relationship:


(9) $$S-S_{0}=-\frac{1}{Y_{G}}\,\left[\frac{X_{0}\,\exp\left(\mu_{m}\,t\right)}{1-\left(\frac{X_{0}}{X_{m}}\right)}-X_{0}\right]+m_{S}$$

$$\left[\left(\frac{X_{m}}{\mu_{m}}\right)\,\ln\left(1-\left(\frac{X_{0}}{X_{m}}\right)\,\left(1-\exp\left(\mu_{m}\,t\right)\right)\right)\right]$$

where S is the reducing sugar concentration (mg⋅L^–1^), *S_0_* is the initial metabolites concentration (mg⋅L^–1^) and t is time (h).

### Establishment of an optimal model of *Trichoderma* strains co-culture

#### Co-culture condition of *Trichoderma* strains in the flask

The co-culture of *Trichoderma* strains was carried out in a 250 mL flask. Each of the four *Trichoderma* strains was grown on PDA for 5 days at 28°C, respectively. The spores were washed with sterile water and the concentration was adjusted to 2 × 10^8^ spore/ml. To prepare the seed co-culture, 0.5 mL spore suspensions of each *Trichoderma* strain were mixed and inoculated into a 100 mL PD medium and incubated for 2 days at 28°C and 200 rpm in the incubator (ZWY-2112B, Zhicheng, China). Seed co-culture of *Trichoderma* strains of 0.03 % (v/v) was inoculated into a 100 mL sterilized fermentation medium, The co-culture conditions were fixed at 37°C and 120 rpm for different long-term periods. During the fermentation time, biomass was analyzed by examining the culture every 24 h intervals.

#### Selection of carbon sources

To accurately determine the optimal carbon sources, glucose, seaweed residue, yeast powder, lactose, maltose, sucrose, molasses, corn flour, and potato flour were compared to understand the effect of various carbon sources on the co-culture of *Trichoderma* strains in the fermentation medium (100 mL). Each carbon source was added into the co-culture medium at 5% (m/v). The co-culture of four *Trichoderma* strains containing individual carbon sources was made using the above described medium and supplemented with 0.03 % (v/v) seed co-culture was placed in a rotary shaker at 120 rpm and 28°C for 5 days. After 5 days, the test tube cucumber seedling plantlet was used to evaluate co-culture quality in plant promotion. Each treatment was repeated five times.

#### Selection of nitrogen sources

To investigate the effect of organic nitrogen source and inorganic nitrogen source on the co-culture of *Trichoderma* in fermentation medium (100 mL). The organic nitrogen source and carbon source in the original medium were provided by corn flour. On this basis, 2% yeast extract, beef extract, peptone, and soybean protein powder were added, respectively. The sodium nitrate and ammonium sulfate were selected as the inorganic nitrogen source in the original medium. A total of 1% ammonium sulfate, sodium nitrate, and potassium nitrate were used to replace the inorganic nitrogen source in the original medium. A total of 0.03 % seed culture was supplemented with the medium and the flasks were incubated at 120 rpm and 28°C for 5 days. After 5 days, the cucumber seedlings were estimated as described earlier. Each treatment was repeated five times.

#### Selection of inorganic salts and tryptophan

To investigate the effect of inorganic salts on the co-culture of *Trichoderma* strains in a fermentation medium (100 mL). The inorganic salt in the original fermentation medium was replaced by 0.05% sodium chloride, magnesium chloride, potassium dihydrogen phosphate, potassium dihydrogen phosphate, magnesium sulfate heptahydrate, ferrous sulfate heptahydrate, manganese sulfate, and zinc sulfate, respectively. To investigate the effect of tryptophan on the co-culture of *Trichoderma* strains in a fermentation medium (100 mL). A total of 1.0 % tryptophan was added to the original fermentation medium. A total of 0.03 % seed co- culture was supplemented with the medium and the flasks were incubated at 120 rpm and 28°C for 5 days. After 5 day, the test tube cucumber seedlings plantlet was applied to evaluate the co-culture quality in plant growth promotion as described earlier. Each treatment was repeated five times.

#### Plackett-burman design

An 8-factor-15-run Plackett-Burman design experiment was conducted (Table 1). The selection of low (-1) and high (1) levels for each factor (Table 1) was based on previous literature. Five replicates per run were performed when conducting the experiments. A total of 75 flasks were randomly assigned to 15 runs. For statistical modeling, the first-order polynomial linear model shown in Equation (10) was employed.


(10) $$\text{Y}=\beta_{0}\sum \beta_{n}x_{n}\left(n=1,2,\ldots ,k\right)$$

**TABLE 1:** Cucumber hypocotyl length for 15 runs with 8 factors in Plackett–Burman design.

Run^#^	Corn flour (g⋅L^–^^1^)	Glucose (g⋅L^–^^1^)	K_2_HPO_4_ (g⋅L^–^^1^)	FeSO_4_⋅7H_2_O (g⋅L^–^^1^)	(NH_4_)_2_SO_4_ (g⋅L^–^^1^)	Seaweed residue (g⋅L^–^^1^)	Tryptophan (g⋅L^–^^1^)	Beef extract (g⋅L^–^^1^)	Cucumber hypocotyl length (cm)
1	40 (1)	20 (−1)	1.0 (1)	0.4 (−1)	0.5 (−1)	40 (−1)	1.5 (1)	25 (1)	9.95 ± 0.31f
2	40 (1)	40 (1)	0.6 (−1)	0.6 (1)	0.5 (−1)	40 (−1)	0.5 (−1)	25 (1)	7.93 ± 0.21i
3	20 (−1)	40 (1)	1.0 (1)	0.4 (−1)	1.5 (1)	40 (−1)	0.5 (−1)	15 (−1)	12.33 ± 0.14c
4	40 (1)	20 (−1)	1.0 (1)	0.6 (1)	0.5 (−1)	60 (1)	0.5 (−1)	15 (−1)	13.75 ± 0.12a
5	40 (1)	40 (1)	0.6 (−1)	0.6 (1)	1.5 (1)	40 (−1)	1.5 (1)	15 (−1)	5.57 ± 0.21k
6	40 (1)	40 (1)	1.0 (1)	0.4 (−1)	1.5 (1)	60 (1)	0.5 (−1)	25 (1)	10.22 ± 0.11ef
7	20 (−1)	40 (1)	1.0 (1)	0.6 (1)	0.5 (−1)	60 (1)	1.5 (1)	15 (−1)	6.84 ± 0.3j
8	20 (−1)	20 (−1)	1.0 (1)	0.6 (1)	1.5 (1)	40 (−1)	1.5 (1)	25 (1)	11.44 ± 0.12d
9	20 (−1)	20 (−1)	0.6 (−1)	0.6 (1)	1.5 (1)	60 (1)	0.5 (−1)	25 (1)	13.04 ± 0.17b
10	40 (1)	20 (−1)	0.6 (−1)	0.4 (−1)	1.5 (1)	60 (1)	1.5 (1)	15 (−1)	8.93 ± 0.25h
11	20 (−1)	40 (1)	0.6 (−1)	0.4 (−1)	0.5 (−1)	60 (1)	1.5 (1)	25 (1)	5.85 ± 0.18k
12	20 (−1)	20 (−1)	0.6 (−1)	0.4 (−1)	0.5 (−1)	40 (−1)	0.5 (−1)	15 (−1)	13.9 ± 0.2a
13	30 (0)	30 (0)	0.8 (0)	0.5 (0)	1.0 (0)	50 (0)	1.0 (0)	20 (0)	9.55 ± 0.19g
14	30 (0)	30 (0)	0.8 (0)	0.5 (0)	1.0 (0)	50 (0)	1.0 (0)	20 (0)	9.21 ± 0.24gh
15	30 (0)	30 (0)	0.8 (0)	0.5 (0)	1.0 (0)	50 (0)	1.0 (0)	20 (0)	10.54 ± 0.37e

Eight factors were shown as real values (coded levels). Cucumber hypocotyl length shown as mean (standard deviation) (n = 5). The selection of low (−1) and high (1) levels for each factor was based on previous literature. Different lowercase letters indicate the significant differences between the treatments by using Tukey’s HSD test.

where Y is the predicted cucumber hypocotyl length (cm), β_0_ is the model intercept, β_*n*_ is the linear coefficient and *x_n_* is the coded level of the independent variable. The main effects plot for Plackett–Burman Design was obtained using “Design-Expert” software (version 12.0, Stat-Ease, Inc., USA). A significant level was set as a *p*-value <0.05. The significant independent variables were considered for further optimization using a central composite design. Because not all components were essential nutrients for fermentation, non-significant components may be removed. Follow-up experiments were carried out to observe if the non-significant variables could be removed.

#### Path of steepest ascent method

The response surface fitting equation is established depending on the optimal level factors approach. And the optimum level scope of the key factors was examined by the path of steepest ascent method (Yang et al., 2017). The climbing direction of the steepest ascent method (Table 2) was determined by the positive and negative effects of the key factors according to the results of Plackett–Burman design.

**TABLE 2:** Steep climbing test design and results.

Run^#^	Glucose (g⋅L^–^^1^)	corn flour (g⋅L^–^^1^)	K_2_HPO_4_ (g⋅L^–^^1^)	Tryptophan (g⋅L^–^^1^)	Cucumber hypocotyl length (cm)
1	30	20	0.5	1.0	12.04 ± 0.12e
2	24	18	0.9	0.8	13.48 ± 0.21d
3	18	16	1.0	0.6	16.18 ± 0.51c
**4**	**12**	**12**	**1.1**	**0.4**	**18.21 ± 0.14a**
5	6	10	1.2	0.2	17.58 ± 0.24b

The bold type indicates the conditions that resulted in the longest cucumber hypocotyl length. Different lowercase letters indicate the significant differences between the treatments by using Tukey’s HSD test.

#### Central composite design

The central composite design (CCD) was performed to determine the optimum factors. Independent variable levels and design structure were shown in Table 3. The selection of five levels of four independent variables was based on results from the path of steepest ascent method (Table 2). A 4-factor-31-run CCD experiment was performed, and five replicates per run were performed when conducting the experiments. A total of 155 flasks were randomly placed in two identical incubators. The effect of the incubator was found to be non-significant (*p*-value < 0.05) and was removed from the model. The polynomial quadratic model shown in Equation (11) was employed to fit the experimental data.


(11) Y=b0⁢∑bn⁢Xn⁢∑b⁢Xn2n⁢n⁢∑β⁢Xnn⁢m⁢Xm

(n,m=1,2,…,k)

**TABLE 3:** The central composite design structure and experimental cucumber hypocotyl length.

Run^#^	Factors				Cucumber hypocotyl length (cm)
	
	Glucose (g⋅L^–^^1^)	Corn flour (g⋅L^–^^1^)	K_2_HPO_4_ (g⋅L^–^^1^)	Tryptophan (g⋅L^–^^1^)	
1	(−1)	10 (−1)	1.0 (−1)	0.2 (−1)	17.85 ± 0.31bc
2	18 (1)	10 (−1)	1.0 (−1)	0.2 (−1)	15.21 ± 0.24jk
3	6 (−1)	26 (1)	1.0 (−1)	0.2 (−1)	17.02 ± 0.36f
4	18 (1)	26 (1)	1.0 (−1)	0.2 (−1)	14.99 ± 0.18kl
5	6 (−1)	10 (−1)	1.2 (1)	0.2 (−1)	18.45 ± 0.12a
6	18 (1)	10 (−1)	1.2 (1)	0.2 (−1)	15.55 ± 0.15ij
7	6 (−1)	26 (1)	1.2 (1)	0.2 (−1)	18.02 ± 0.11b
8	18 (1)	26 (1)	1.2 (1)	0.2 (−1)	15.52 ± 0.25ij
9	6 (−1)	10 (−1)	1.0 (−1)	0.6 (1)	15.04 ± 0.13kl
10	18 (1)	10 (−1)	1.0 (−1)	0.6 (1)	13.88 ± 0.16p
11	6 (−1)	26 (1)	1.0 (−1)	0.6 (1)	14.58 ± 0.34mno
12	18 (1)	26 (1)	(−1)	0.6 (1)	13.48 ± 0.28q
13	6 (−1)	10 (−1)	1.2 (1)	0.6 (1)	15.65 ± 0.16hi
14	18 (1)	10 (−1)	1.2 (1)	0.6 (1)	14.22 ± 0.21op
15	6 (−1)	26 (1)	1.2 (1)	0.6 (1)	15.26 ± 0.17jk
16	18 (1)	26 (1)	1.2 (1)	0.6 (1)	13.95 ± 0.24p
17	0 (−2)	18 (0)	1.1 (0)	0.4 (0)	17.96 ± 0.23b
18	24 (2)	18 (0)	1.1 (0)	0.4 (0)	14.47 ± 0.12no
19	12 (0)	2 (−2)	1.1 (0)	0.4 (0)	15.74 ± 0.21hi
20	12 (0)	34 (2)	1.1 (0)	0.4 (0)	14.92 ± 0.16klm
21	12 (0)	18 (0)	0.9 (−2)	0.4 (0)	14.66 ± 0.18lmn
22	12 (0)	18 (0)	1.3 (2)	0.4 (0)	15.94 ± 0.41h
23	12 (0)	18 (0)	1.1 (0)	0 (−2)	16.52 ± 0.16g
24	12 (0)	18 (0)	1.1 (0)	0.8 (2)	12.63 ± 0.52r
25	12 (0)	18 (0)	1.1 (0)	0.4 (0)	17.22 ± 0.14def
26	12 (0)	18 (0)	1.1 (0)	0.4 (0)	17.45 ± 0.13ed
27	12 (0)	18 (0)	1.1 (0)	0.4 (0)	17.09 ± 0.12ef
28	12 (0)	18 (0)	1.1 (0)	0.4 (0)	17.47 ± 0.18cde
29	12 (0)	18 (0)	1.1 (0)	0.4 (0)	17.54 ± 0.35cd
30	12 (0)	18 (0)	1.1 (0)	0.4 (0)	17.35 ± 0.26def
31	12 (0)	18 (0)	1.1 (0)	0.4 (0)	17.52 ± 0.17cd

Four factors were shown as real values (coded levels). Cucumber hypocotyl length were shown as mean (standard deviation) of 5 replicates for run #1–15. For experimental levels for cube (−1 and 1) and star (−2 and 2) points were set around the experimental levels of center points. Different lowercase letters indicate the significant differences between the treatments by using Tukey’s HSD test.

where Y is the predicted cucumber hypocotyl length (cm); *b_0_* is the intercept; *b_n_* is the linear coefficient; *b*_*nn*_ is the quadratic coefficient and *b*_*nm*_ is the interaction coefficient. Xn and Xm are independent variables.

The model in Equation (11) was fit to experimental data generated from *b*_*nm*_ the central composite design using “Design-Expert” software. The fitted model was evaluated based upon the model sufficiency (*R*^2^), regression parameter significance (*P*-value), and test for lack of fit. *P*-values < 0.05 was set as statistically significant.

The optimal medium concentration of prediction was determined by the response surface optimizer, and five groups of parallel experiments under this optimal medium were conducted to analyze whether the actual results are within the 95% confidence interval of the predicted value, to verify the accuracy of the model prediction.

### Statistical analysis

All the measurements were done in at least triplicate, and the data were presented in the average values ± standard deviation. Analysis of variance (ANOVA) was conducted using IBM SPSS Statistics version 23 (IBM, USA), and *P* < 0.05 was considered to be significant. The Origin 2022b (OriginLab, USA) was used for kinetic model fitting. GraphPad Prism 8 (GraphPad Software, USA) was used for data visualization.

## Results

### Selection of inoculation technology for high plant promotion of co-culture metabolites from *Trichoderma* strains

**FIGURE 1:**
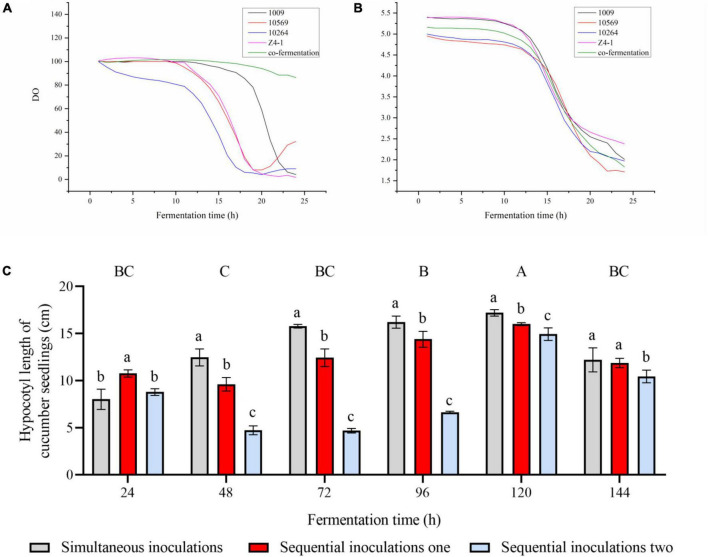
Effect of inoculation technology on the bioreactor. **(A)** The dynamic curve of pH of *Trichoderma* co-fermentation broth in 50 L bioreactor. **(B)** The dynamic curve of dissolved oxygen (DO) in *Trichoderma* fermentation broth in 50 L bioreactor. **(C)** Effect of fermentation broth with different inoculation sequences on the growth of cucumber plantlets *in vitro*. Results are expressed as mean of five repeats, vertical bar represents standard error of the mean at significant (*P* < 0.05) using Tukey’s HSD test. Different uppercase letters indicate the significant differences between time and lowercase letters indicates significant differences between treatments in different time intervals.

The sequential inoculation and simultaneous inoculation were compared in this study. In the axenic culture, the pH was no significant change at lag phase of 0–10 h, and pH drops rapidly at the logarithmic phase of 10–20 h (Figure 1A). Therefore, it is necessary to accomplish the inoculation before the pH enters the logarithmic phase to avoid low pH affecting *Trichoderma* spore germination. In co-culture, sequential inoculation 1 is mainly based on pH changes, with strain 10569 inoculated firstly, followed by strain 10264 after 8 h, then strain 1009 and z4-1 were inoculated after 10 h.

To avoid the influence of dissolved oxygen (DO) on the growth of *Trichoderma* (Figure 1B), in axenic culture, the lag phase of DO was 0–10 h and the logarithmic period at 10–20 with a rapid decline in DO. Therefore, the inoculation should be completed before the DO reaches the logarithmic phase, to avoid low DO affecting *Trichoderma* spore germination. In co-culture, sequential inoculation 2 is mainly accomplished following DO changes, where strain 1009 was inoculated firstly, followed by strain 10569 and strain z4-1 after 5 h, and strain 10264 was inoculated after 8 h.

To compare the effect of simultaneous inoculation and sequential inoculation in the co-culture of four *Trichoderma* strains, the fermentation metabolites were sampled every 24 h until the pH dropped to 2.3 (Figure 1C). The co-culture metabolites from simultaneous inoculation were lower in cucumber seedling hypocotyl growth than that of sequential inoculation at 24 h, however, the fermentation metabolites were far from completely produced at this time. In other time points of co-culture inoculation, the co-cultures from simultaneous inoculation generated higher cucumber seedling hypocotyl growth promotion than that of the co-culture metabolites from sequential inoculation, indicating that simultaneous inoculation is better than sequential inoculation in terms of plant growth promotion with co-culture metabolites, and further sequential inoculation 1 is better than sequential inoculation 2.

### Selection of the optimal fermentation time for co-culture of *Trichoderma* strains

**FIGURE 2:**
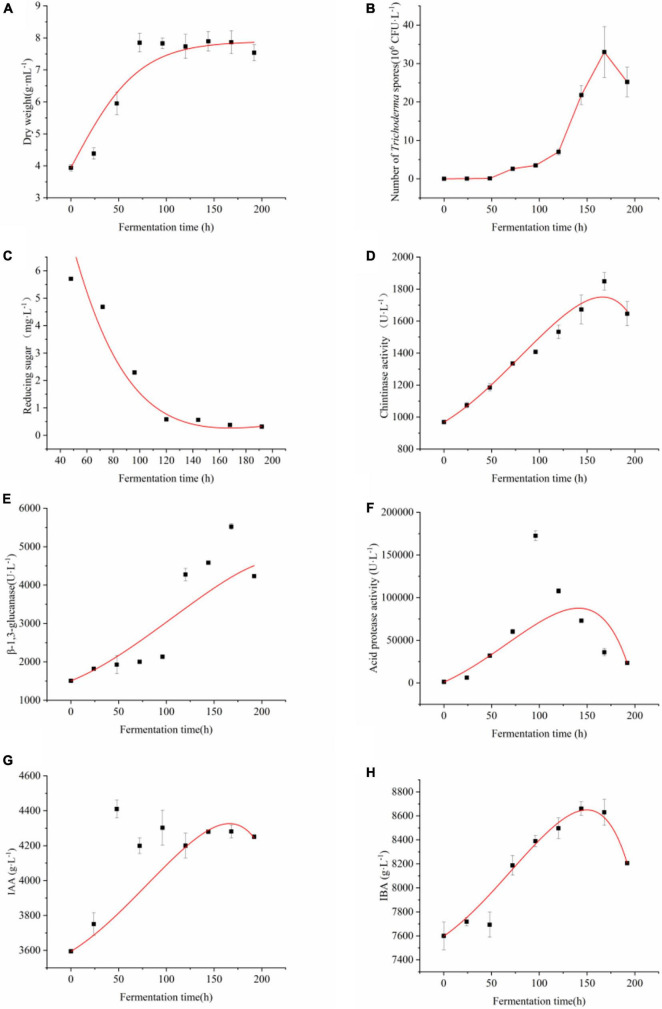
Time-course of the batch culture of four *Trichoderma*. Changes of **(A)** dry weight of *Trichoderma* **(B)** number of viable conidial spores of Trichoderma, **(C)** reducing sugar content, **(D)** chitinase productionβ-1,3-glucanase production, **(E)** β-1,3-glucanase production, **(F)** acid protease production, **(G)** IAA production, and **(H)** IBA production during the co-fermentation process of four *Trichoderma*. The experiments were done in quintuplicate. The error bars were represented in the graph; standard deviation and mean were determined.

Figure 2 illustrates the time-course of the co-culture of four *Trichoderma* strains in the medium containing components as follows: Corn flour 50 g/L, KH_2_PO_4_ 3.82 g/L, NaNO_3_ 1.42 g/L, (NH_4_)_2_SO_4_ 1.1 g/L, NaCl 1 g/L, MgSO_4_⋅7H_2_O 0.5 g/L, FeSO_4_⋅7H_2_O 0.0075 g/L, MnSO_4_ 0.0025 g/L, ZnSO_4_ 0.002 g/L. The total liquid volume of the medium was 35 L. The kinetic parameters for biomass growth, antimicrobial substances, and plant growth regulators production, glucose consumption at different speeds of agitation were shown in Table 4. The growth of biomass was separated into three stages, as follows:

**TABLE 4:** Kinetic parameters for bioreactor cultivation.

Parameter	IAA	IBA	Chitinase	β-1,3-glucanase	Acid protease
*X*_0_ (g⋅L^–1^)	3.942 ± 0.103
*X*_m_ (g⋅L^–1^)	7.895 ± 0.305
μ_m_ (g⋅h^–1^)	0.028 ± 0.005
α	−0.274 ± 0.068 (g⋅L^–1^)	−0.734 ± 0.043 (g⋅L^–1^)	−0.295 ± 0.089 (U⋅L^–1^)	−0.199 ± 0.587 (U⋅L^–1^)	−84.563 ± 6.00 (U⋅L^–1^)
β	0.865 ± 0.065 (g⋅L^–1^)	1.459 ± 0.058 (g⋅L^–1^)	0.925 ± 0.020 (U⋅L^–1^)	2.537 ± 0.581 (U⋅L^–1^)	132.024 ± 6.720 (U⋅L^–1^)
*Y*_G_ (mg⋅g^–1^)	0.193 ± 0.004
m_S_ (mg⋅g⋅h^–1^)	0.00126 ± 0

IAA means Indole-3-acetic acid and IBA indole-3-butyric acid. ± 95 % confidence interval.

Stage I is 0–48 h.

A 48-h exponential phase with maximum dry weight growth rate (0.028 ± 0.005 g⋅h^–1^) of *Trichoderma* was observed. In this phase, the yield of antimicrobial, cucumber growth promotion metabolites started to accumulate. And the conidial spores of *Trichoderma* were produced minimally.

Stage II: 72–144 h.

After the initial accumulation, the conidial spores of *Trichoderma* began to grow with a linear increasing specific growth rate (Figure 2B) after 48 h. Evident accumulation of chitinase, β-1,3-glucanase, acid protease activity, IAA, and IBA began after 48 h. Acid protease (Figure 2F) and IBA (Figure 2H) reached the highest of 73007.776 U⋅L^–1^ and 8700 g⋅L^–1^, respectively.

Stage III: 144–196 h.

*Trichoderma* growth was inhibited and substrate consumption stopped in this phase. Maximum biomass (number of viable spores) of 3.3 × 10^7^ CFU⋅mL^–1^ was achieved at 168 h (Figure 2B), growth was slowed down afterward, and chlamydospore was accounted for the majority in this time. The chitinase (Figure 2D), β-1,3-glucanase (Figure 2E) activity, and IAA content (Figure 2G) reached highest in this phase, respectively.

**FIGURE 3:**
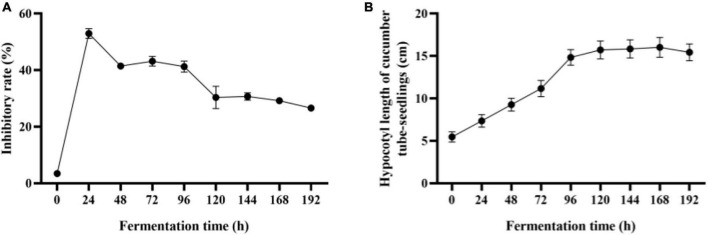
Batch co-culture of *Trichoderma* composite in the bioreactor. **(A)** Inhibition effect of sterile filtrate of co-fermentation broth against *F. oxysporum*. **(B)** Growth-promoting effects (cucumber hypocotyl length) of co-fermentation broth. The experiments were done in quintuplicate. The error bars were represented in the graph; standard deviation and mean were determined.

With the increase of mycoparasitism-related enzyme activity, *Trichoderma* co-culture metabolites inhibition to *F. oxysporum* reached the highest of 52.92 % in 24 h, and the inhibitory rate remained above 40% within 96 h (Figure 3A). As the co-culture continued, mycoparasitism-related enzyme activities were still being increased, but co-culture broth inhibitory effect to *F. oxysporum* growth was compromised to about 30 %. To achieve the ideal plant growth promotion effect with co-culture broth (Figure 3B), co-culture at 0–120 h enable to make metabolites with increased plant promotion effect. Afterward, the effect for plant promotion with co-culture turn to a stationary stage. The growth-promoting ability of co-culture metabolites was found to match the growth of *Trichoderma* and partially matched the IAA and IBA production in co-culture. This study elucidated that 96 to 120 h co-culture was suitable for the production of metabolites with high *F. oxysporum* inhibitory effect and plant growth promotion activity.

Similar biomass values were detected in the stationary phase of batch fermentations with different glucose concentrations, which indicated that the antimicrobial and cucumber growth promotion metabolites fermentation process follow the classical kinetic model of substrate-limited biomass growth (Monod, 1949). The kinetic parameters for biomass growth (dried weight), plant growth promotion and antagonism metabolites production and glucose consumption are shown in Table 4. The maximum specific growth rate (μ_*m*_ = 7.895 ± 0.305) in Table 4 were calculated by fitting the Equation (2) to the measured fermentation profiles. The model predicted biomass yield coefficient on glucose (*Y_G_*) and the cell maintenance coefficient on glucose (*m_s_*) were 0.193 ± 0.004 and 0.00126 ± 0, respectively.

According to the relationship between product formation and cell growth, product formation was divided into three categories: a. product formation is coupled with cell growth; b. Product formation is partially coupled with cell growth; c. There is no relationship between product formation and cell growth. Luedeking-Piret (Equation 4) can be used to describe the relationship between product formation and cell growth in this study (Saat et al., 2013). The IAA, IBA, chitinase, β-1,3-glucanase, and acid protease activity formed a partial growth coupling type and α≠ 0, β≠ 0 (Figure 2). The growth-associated constant (α) of IAA, IBA, chitinase, β-1,3-glucanase, and acid protease activity were −0.274 ± 0.068, −0.734 ± 0.043, −0.295 ± 0.089, −0.199 ± 0.587, −84.563 ± 6.00, respectively. The non-growth-associated constant (β) were 0.865 ± 0.065, 1.459 ± 0.058, 0.925 ± 0.020, 2.537 ± 0.581, 132.024 ± 6.720, respectively.

### Effect of medium components on cucumber seedling growth

**FIGURE 4:**
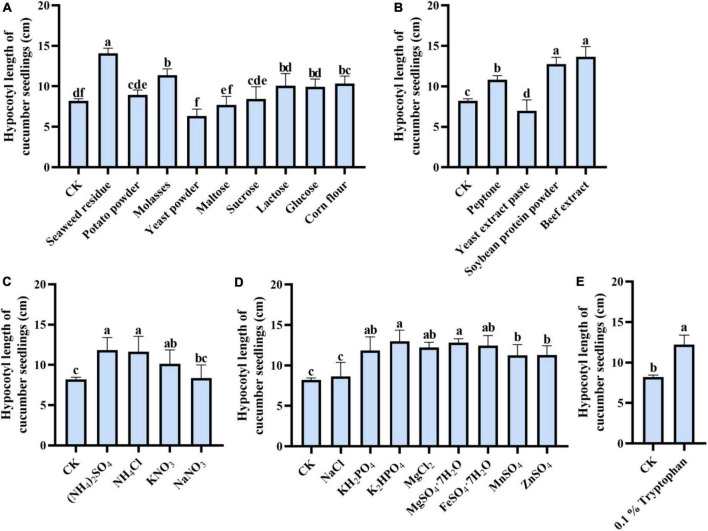
One-factor-in-a-time method for the significant medium component screen. **(A)** Each of various carbon sources, **(B)** each of various organic nitrogen sources, **(C)** each of various inorganic nitrogen sources, **(D)** each of various inorganic salts **(E)** 1 % tryptophan on the growth-promoting ability of co-fermentation broth. The cucumber hypocotyl length and the response value were represented, respectively. The experiments were done in quintuplicate. The error bars were represented in the graph; standard deviation and mean were determined (*P* < 0.05) using Tukey’s HSD test. Different lowercase letters indicate the significant differences between the treatments.

By comparing the effects of different carbon sources in the medium on the metabolite’s performance in the promotion of cucumber, we found that the *Trichoderma* strain’s co-culture metabolites had different promotion effects on cucumber seedling growth (Figure 4), in which the seaweed residue as a single carbon source in the medium exerted a maximum effect on cucumber hypocotyl growth (14.07 cm), others were followed by potato powder (9.28 cm), molasses (11.38 cm), yeast powder (6.32 cm), maltose (7.7 cm), sucrose (8.44), lactose (10.06 cm), and glucose (9.92 cm) (Figure 4A).

The effect of different nitrogen in medium on co-culture metabolites function to promote cucumber seedling growth was compared. The cucumber seedling growth promotion effect of nitrogen (Figures 4B,C) in the medium for co-culture was shown from high to low as follow: beef extract (13.65 cm), soybean protein powder (12.76 cm), ammonium sulfate (11.83 cm), and ammonium chloride (11.64 cm). However, the effects of ammonium chloride and soybean were not significant. And further, it was revealed that the co-culture metabolites induced by tryptophan were more significant in the promotion of cucumber seedling hypocotyl growth, in which the length of hypocotyl reached 12.2 cm.

Moreover, the biosynthesis of metabolites also needs some salt. In this study, dipotassium hydrogen phosphate and ferrous sulfate heptahydrate as the medium components can induce more production of microbial metabolites required for the growth of cucumber seedlings (Figures 4D,E).

### Optimized design and experiment for medium components formulation

The Plackett-Burman design (PBD) was carried out with 8 factors as variables: corn flour, glucose, dimethyl hydrogen phosphate, ferrous sulfate heptahydrate, ammonium sulfate, seaweed residue, tryptophan, and beef paste. Since the metabolites produced in the original medium only made cucumber hypocotyl length 8.20 cm, it was necessary to screen the medium component for the production of more quality co-culture metabolites. The significant components were screened under the PBD, the cucumber hypocotyl length is shown in Table 1. After fitting a first-order polynomial model, the coefficient of determination (*R*^2^) was 0.9862 (Table 1), which indicated a good fit. Four components, tryptophan, glucose, dipotassium hydrogen phosphate, and corn flour were found to have more significant effects on cucumber hypocotyl length than those from the original medium (Table 5).

**TABLE 5:** Analysis of variance (ANOVA) for 8-run Plackett–Burman design and model fitting.

Source	DF	Sum of squares	Mean square	*F*-value	*P*-value
Model	9	98.0217	10.8913	39.85	0.000**
Corn flour	1	4.1419	4.1419	15.15	0.011*
Glucose	1	41.3294	41.3294	151.21	0.000**
K_2_HPO_4_	1	7.2230	7.2230	26.43	0.004**
FeSO_4_⋅7H_2_O	1	0.5677	0.5677	2.08	0.209
(NH_4_)2SO_4_	1	0.9130	0.9130	3.34	0.127
Seaweed residue	1	0.5167	0.5167	1.89	0.228
Tryptophan	1	42.5257	42.5257	155.59	0.000**
Beef extract	1	0.6960	0.6960	2.55	0.171
Lack-of-Fit	3	0.4118	0.1373	0.29	0.835
Pure error	2	0.9549	0.4774		
Total	14	99.3883			

R^2^ = 0.9862; R^2^_Adj_ = 0.9615; R^2^_prediction_ = 0.9121. **P < 0.01 and *P < 0.05.

Follow-up experiments were conducted to observe whether cucumber hypocotyl length was affected if removing non-significant factors. First, seaweed residue (nitrogen source) was removed from the medium, cucumber hypocotyl length was dropped to 7.76 ± 0.20 cm, significantly (*P* < 0.01) different from 13.9 ± 0.20 cm (cucumber hypocotyl length of run #12 in Table 1). The removal of the beef extract (7.64 ± 0.67 cm) has no significant impact on the growth of cucumber hypocotyl compared to the removal of seaweed residue. In consideration of the higher cost of beef extract, it was removed from the medium.

Ammonium sulfate and ferrous sulfate heptahydrate were also found to be non-significant factors from Plackett-Burman design. However, after removing seaweed residue and ferrous sulfate heptahydrate, cucumber hypocotyl length was decreased to 7.04 ± 0.57 and 6.96 ± 0.49 when removing, respectively. Removal of seaweed residue and ferrous sulfate heptahydrate in the medium led to co-culture metabolites being less effective to stimulate cucumber hypocotyl length, which implied it was necessary to keep seaweed residue and ferrous sulfate heptahydrate in the medium for the production of quality co-culture metabolites with high plant promotion activities.

To further evaluate the comprehensive effects of tryptophan, glucose, dipotassium hydrogen phosphate, and corn flour on cucumber seedling hypocotyl growth, the path of steepest ascent method was used to determine the center point of the response surface. It was evident (Table 2) that the optimal cucumber hypocotyl length reached the highest peak at the fourth step where the concentrations of tryptophan, glucose, dipotassium hydrogen phosphate, and corn flour reached 0.4, 12, 1.1, and 12 g⋅L^–1^, respectively.

To determine the interaction of tryptophan, glucose, dipotassium hydrogen phosphate, and corn flour, the central composite design was applied to estimate the interaction effects of the four factors on the cucumber seedling growth. The longest cucumber hypocotyl length was obtained at the center point (run #5) (Table 3), A significant model (*p* < 0.0001) with a non-significant lack of fit (*P* = 0.486) and an R-sq of 0.9878 was obtained (Table 6). the model [Equation (11)] was adequate to fit the experimental data.


$$\begin{array}{l} cucumberhypocotyllength\;\;=\;\;-46.15\;+\;0.0249Glucose+\\ \;\;\;\;\;\;\;\;\;\;\;\;\;\;\;\;\;\;\;\;\;\;\;0.1597conflour\\ \;\;\;\;\;\;\;\;\;\;\;\;\;\;\;\;\;\;\;\;\;\;\;+111.23K_{2}HPO_{4}+\\ \;\;\;\;\;\;\;\;\;\;\;\;\;\;\;\;\;\;\;\;\;\;\;\;6.43Tryptophan\\ \;\;\;\;\;\;\;\;\;\;\;\;\;\;\;\;\;\;\;\;\;\;\;-0.007205Glucose*Glucose\\ \;\;\;\;\;\;\;\;\;\;\;\;\;\;\;\;\;\;\;\;\;\;\;-0.007510Cornflour*Cornflour\\ \;\;\;\;\;\;\;\;\;\;\;\;\;\;\;\;\;\;\;\;\;\;\;-48.81K_{2}HPO_{4}^{*}K_{2}HPO_{4}\\ \;\;\;\;\;\;\;\;\;\;\;\;\;\;\;\;\;\;\;\;\;\;\;-16.735Tryptophan^{*}\\ \;\;\;\;\;\;\;\;\;\;\;\;\;\;\;\;\;\;\;\;\;\;\;\;Tryptophan\\ \;\;\;\;\;\;\;\;\;\;\;\;\;\;\;\;\;\;\;\;\;\;\;+0.001549Glucose^{*}Cornflour\\ \;\;\;\;\;\;\;\;\;\;\;\;\;\;\;\;\;\;\;\;\;\;\;-0.1260Glucose^{*}K_{2}HPO_{4}\\ \;\;\;\;\;\;\;\;\;\;\;\;\;\;\;\;\;\;\;\;\;\;\;+0.2641Glucose^{*}Tryptophan\\ \;\;\;\;\;\;\;\;\;\;\;\;\;\;\;\;\;\;\;\;\;\;\;+0.0617Cornflour^{*}K_{2}HPO_{4}\\ \;\;\;\;\;\;\;\;\;\;\;\;\;\;\;\;\;\;\;\;\;\;\;-0.0004Cornflour^{*}Tryptophan\\ \;\;\;\;\;\;\;\;\;\;\;\;\;\;\;\;\;\;\;\;\;\;\;-1.16K_{2}HPO_{4}{}^{*}Tryptophan \end{array}$$

**TABLE 6:** Regression results of the CCD and ANOVA for quadratic model.

Source	Sum of squares	df	Mean square	*F*-value	*p*-value
Model	72.00	14	5.14	174.66	<0.0001
A-Glucose	20.26	1	20.26	688.01	<0.0001
B-Corn flour	0.9087	1	0.9087	30.86	<0.0001
C-Dipotassium hydrogenphosphate	2.12	1	2.12	71.94	<0.0001
D-Tryptophan	24.66	1	24.66	837.65	<0.0001
A × B	0.0885	1	0.0885	3.01	0.1022
A × C	0.0915	1	0.0915	3.11	0.0970
A × D	1.61	1	1.61	54.56	<0.0001
B × C	0.0390	1	0.0390	1.32	0.2667
B × D	6.250 E-06	1	6.250 E-06	0.0002	0.9886
C × D	0.0086	1	0.0086	0.2906	0.5973
A^2^	1.92	1	1.92	65.34	<0.0001
B^2^	6.61	1	6.61	224.35	<0.0001
C^2^	6.81	1	6.81	231.41	<0.0001
D^2^	12.81	1	12.81	435.16	<0.0001
Residual	0.4711	16	0.0294		
Lack of fit	0.3024	10	0.0302	1.08	0.4864
Pure error	0.1687	6	0.0281		
Cor total	72.47	30			

**FIGURE 5:**
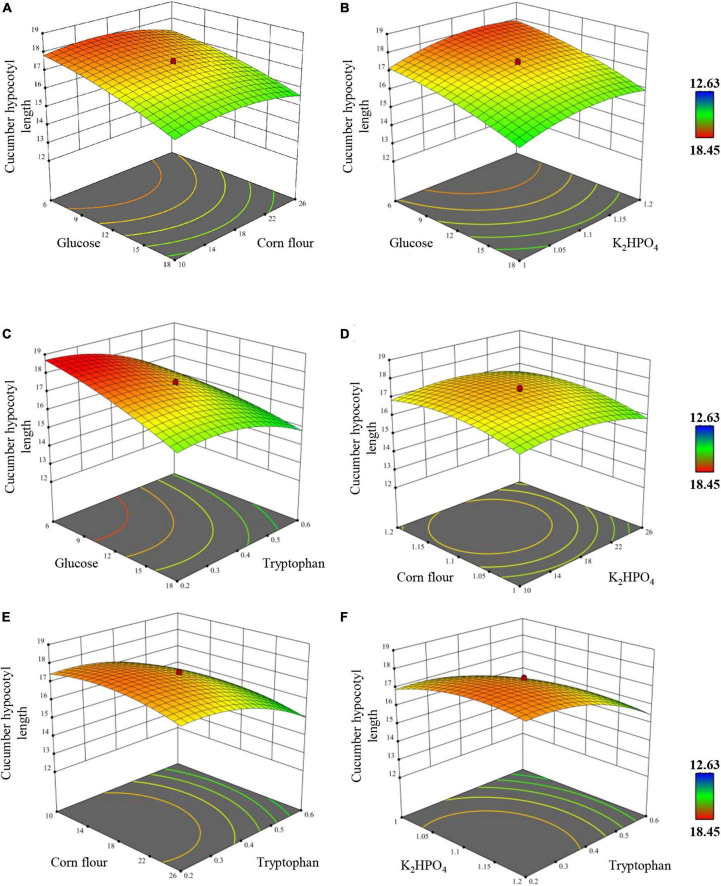
Response surface and contour plot exhibiting the overall effects of **(A)** corn flour vs. glucose, **(B)** K_2_HPO_4_ vs. glucose, **(C)** tryptophan vs. glucose, **(D)** K_2_HPO_4_ vs. corn flour, **(E)** tryptophan vs. corn flour, **(F)** tryptophan vs. K_2_HPO_4_ on the hypocotyl length of cucumber plantlets *in vitro*. Only tryptophan vs. glucose interaction is significant at *p*-value of <0.0001.

The overall effects were exhibited by plotting two factors as the independent variables and cucumber hypocotyl length as the dependent variable with the four factors fixed at their optimum level (Figure 5). All four factors showed a clear quadratic effect, which was consistent with the significant effects (*p* < 0.0001) of quadratic terms in Table 6. Finally, the regression mode was established as *Y* = 17.3771–0.9188 A–0.1946 B + 0.2971 C–1.0138 D–0.2594 A × A–0.4806 B × B −0.4881 C × C–0.6694 D × D + 0.3169 A × D, the model coefficient determination was determined as R-sq = 99.04 %, R-sq (Adj) = 98.62 %, R-sq (prediction) = 97.45 %. Based on the model, a highly significant interaction of tryptophan × tryptophan, glucose × glucose, dipotassium hydrogen phosphate × dipotassium, corn flour × corn flour, and tryptophan × glucose was revealed (*p* < 0.0001), while others interaction is insignificant (*p* > 0.05), indicating that all factors response values were not simple linear regression relation, instead, a kind of partial quadratic regression relation.

### Optimum medium concentration and validation

**FIGURE 6:**
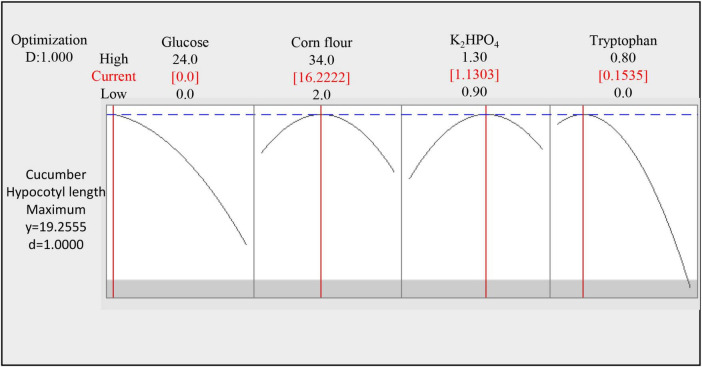
Optimal medium concentration of four significant factors selected by response surface methodology.

According to the central composite design, the longest cucumber hypocotyl length was predicted to be 19.5 cm under the optimum condition (Glucose 0 g⋅L^–1^, Corn flour 16.22 g⋅L^–1^, dipotassium hydrogen phosphate 1.13 g⋅L^–1^, and tryptophan 0.154 g⋅L^–1^ in Figure 6). The experimental result under the optimum condition showed a cucumber hypocotyl length of 19.19 cm, which was close to and validated the predicted result from CCD. It is extraordinary that the supplement system (Glucose 0 g⋅L^–1^, Corn flour 16.22 g⋅L^–1^, dipotassium hydrogen phosphate 1.13 g⋅L^–1^, tryptophan 0.154 g⋅L^–1^, ammonium sulfate 1.5 g⋅L^–1^, ferrous sulfate heptahydrate 1 g⋅L^–1^, and seaweed residue 30 g⋅L^–1^) improved cucumber hypocotyl length by 2.3-fold from 8.20 to 19.19 cm.

## Discussion

The global scientific community has taken a great interest in *Trichoderma* spp. in many aspects, not only the biocontrol function of Trichoderma living spores, but also the diversified metabolites with multiple functions (Keswani et al., 2013). Natural products (NPs) from *Trichoderma* are considered as the potential fungicide and fertilizer for being applied in agriculture industry. However, one of the major limitations of NP development is that most of discovered compounds was simply yielded from single strain culture, and the majority of components were known compounds. Therefore, in recent years, co-cultivation of various microorganisms was fast developed for the massive production of valuable metabolites, especially focused on inducing novel chemical compounds, which has been commonly seen as a major strategy to address this limitation (Arora et al., 2020).

A key factor in the success of microbial co-culture is inoculation technology. According to previous studies, the sequential inoculation strategy is crucial for the co-culture of *Trichoderma*, particularly for co-culture with incompatible microorganisms. The sequential inoculation has been confirmed one of techniques, which enable to provide a balanced growth and effective interaction among various microorganisms in consortia (Karuppiah et al., 2019; Li et al., 2020). Sequential inoculation for co-culture of *Trichoderma* strains is demonstrated to be an ideal way for gaining the quality co-culture metabolites. Besides, adjusting of the inoculation ratio, culture temperature, pH, etc., is also alternative for balancing the growth in co-culture, for example, by optimizing inoculation ratio between *Trichoderma* and *Aspergillus tubingensis* (Intasit et al., 2021). The co-cultivation of *Trichophyton rubrum* and *T. longibrachiatum* can produce more laccase activity by adjusting the inoculation ratio to 0.5:1 (Jung, 2019). Differently, the four *Trichoderma* strains used in this study can synergistically interact with one another in the fermentation tank for longer periods since those strains were pre-evaluated to be high comparable each other. Hence, it is easy to build a synthetic flora of *Trichoderma* with multi-functional synergistic interaction. This resulted in a much higher activity of plant growth promotion metabolites than when inoculated sequentially.

In previous studies, the co-cultivation of *Trichoderma* and other microorganisms, such as *Trichoderma*-*Bacillus* and *Trichoderma*-*Penicillium*, was thoroughly explored. These co-cultures significantly stimulated the expression of an array of genes related to enzymes, including non-ribosomal peptide synthetase, putative ferrichrome synthetase, polyketide synthetase, Chitinase, β-1,3-glucanase, and acid protease, etc. Some of which were also involved in the regulation of the production of secondary metabolites (Nonaka et al., 2011; Karuppiah et al., 2019; Li et al., 2020). Co-cultivation of *B. amyloliquefaciens* ACCC11060 and *T. asperellum* GDFS1009 was able to produce more specific biocontrol (apigenin) and plant growth promotion (IAA) metabolites (Wu et al., 2018). But there were few studies on the co-culture of various *Trichoderma* strains. Co-culture of *Trichoderma* strains with different characteristics might also produce more specific metabolites than axenic culture in certain condition. In the present study, modeling the kinetic of the co-culture process of *Trichoderma* multiple strains was of great significance to deciding when to collect metabolites along the co-culture process, due to the dynamic changes of metabolites production were happened following the fermentation time. The optimal co-culture for the formation of plant growth promotion related metabolites was at the stage between 96 and 120 h, it did not point to the specific content, structure, or particular species of the numerous metabolites along co-culture.

The chitinase, β-1,3-glucanase, and acid protease were reported to be associated with the mycoparasitism of *Trichoderma*. The production of chitinases, β-1,3-glucanases and proteases facilitates *Trichoderma* to parasitize the host fungi (Mukherjee et al., 2013). The role of *Trichoderma* phytohormone IAA in *Arabidopsis* growth and development was IAA-induced shoot growth promotion and root branching (Contreras-Cornejo et al., 2009). And IBA was reported to be auxin precursor, it can active the synthesis of IAA (Frick and Strader, 2017). This study discovered that there was a certain correlation between the growth of microorganisms and the metabolite synthesis during co-culture. For example, the reducing sugar content is opposite to cell growth, and the yield of chitinase, β-1,3-glucanase, acid protease IAA and IBA were partially related to cell growth. This result is of great significance for real-time monitoring of the synthesis of specific metabolites through microorganism alterations.

Through the one-factor-at-a-time, Plackett-Burman design, and response surface methodology optimization technique (Singh et al., 2017), it is clear that the core components of the co-culture medium of four *Trichoderma* strains are corn flour, tryptophan, glucose, and dipotassium hydrogen phosphate, which have a very significant effect on the hypocotyl length of cucumber. The L-tryptophan was proved to be the precursor of IAA. During the optimization of IAA production by *T. atroviride* found that addition of L-tryptophan to the medium enable to enhance the production of IAA (Gravel et al., 2007). Phosphate is another basic component which is required for the production of phospholipids present in the microbial cell membranes, and for the production of nucleic acids (Singh et al., 2017). A similar situation also occurred in the present study.

Because partial of components duplicated, non-significant components identified from Plackett-Burman design may be removed from the medium. The removal of the beef extract had no significant impact on the production of plant growth promotion related metabolites. Removing seaweed residue was significant affect the production of plant promotion related metabolites. Seaweed is potential substrate for metabolic activation through fermentation (Tan and Lee, 2014). The seaweed residues, usually were characterized with multiple plant promotion ant-stress functions, have been used as component of the medium for bacteria co-culture, which can induce the production of a variety of antimicrobial metabolites (Martelli et al., 2020). Furthermore, ammonium sulfate and ferrous sulfate heptahydrate also affect the production of plant growth promotion metabolites. The cucumber hypocotyl length can be viewed as an indicator to predict optimum co-culture condition if central composite design (CCD) is applied. The experimental result under the optimum condition was basically consistent with the predicted result from CCD. The content of glucose was zero under the optimal conditions, so glucose was also removed. Application of the optimal medium based on core medium (corn flour, tryptophan, and dipotassium hydrogen phosphate) components was able to produce more reliable plant growth promoting and antimicrobial co-culture agents in the future.

The co-culture technique of Trichoderma strains developed in this work offers a quick screening and evaluation approach to optimize medium components combinations. With the medium optimized, a more active microbial metabolites consortia could be achieved underlying the promotion of crop growth and biocontrol against pathogen infection.

## Conclusion

Statistically based experimental designs proved to be valuable tools in optimizing culture medium for the production of four *Trichoderma* strains co-culture metabolites with high effect in the promotion of cucumber seedling growth. The simultaneous inoculation with *Trichoderma* strains consortia in co-culture was confirmed to be better than sequence inoculation for enhancing the promotion effect of co-culture metabolites to cucumber seedling hypocotyl growth. And the 96–120 h fermentation was optimal for effective synergistic production of antimicrobial and cucumber seedling growth promotion metabolites. Plackett-Burman design used in the first step was an efficient approach to screen nutrient factors for effective production of co-culture metabolites with significant promotion of cucumber seedling growth. Then the method of steepest ascent was employed to approach or near the experimental design space. Central composite designs and response surface analysis used in the last steps were useful to determine the optimum levels of the nutrient factors that significantly influence cucumber growth promotion substances production. Finally, the optimized concentrations of medium components are as follows: corn flour 16.22 g⋅L^–1^, potassium hydrogen phosphate 1.13 g⋅L^–1^, tryptophan 0.154 g⋅L^–1^, seaweed residue 30 g⋅L^–1^, ferrous sulfate heptahydrate 1 g⋅L^–1^, and ammonium sulfate 1.5 g⋅L^–1^. The co-culture metabolites fermented from the optimal medium revealed significantly improved cucumber hypocotyl length by 2.3-fold from 8.20 to 19.19 cm. In addition, the inhibition rate of *F. oxysporum*, the growth-promoting effect of cucumber, and the antifungal and growth promoting metabolites in different periods were conducted in the fermenter. Taken together, the results provided co-fermentation strategies for the preparation of microbial metabolites based-biofertilizer with biocontrol and plant promotion function.

## Data availability statement

The original contributions presented in this study are included in the article/supplementary material, further inquiries can be directed to the corresponding author.

## Author contributions

JC, YL, and XW contributed to conception and design of the study. HL and DH organized the database and performed the statistical analysis. HL wrote the first draft of the manuscript. HL and JC wrote sections of the manuscript. All authors contributed to manuscript revision, read, and approved the submitted version.
